# Encoding, transmission, decoding, and specificity of calcium signals in plants

**DOI:** 10.1093/jxb/erac105

**Published:** 2022-03-17

**Authors:** Claudia Allan, Richard J Morris, Claudia-Nicole Meisrimler

**Affiliations:** University of Canterbury, School of Biological Science, Christchurch, New Zealand; Computational and Systems Biology, John Innes Centre, Norwich, UK; University of Canterbury, School of Biological Science, Christchurch, New Zealand; Bielefeld University, Germany

**Keywords:** Calcium signaling, information theory, long-distance signaling, molecular communications

## Abstract

Calcium acts as a signal and transmits information in all eukaryotes. Encoding machinery consisting of calcium channels, stores, buffers, and pumps can generate a variety of calcium transients in response to external stimuli, thus shaping the calcium signature. Mechanisms for the transmission of calcium signals have been described, and a large repertoire of calcium binding proteins exist that can decode calcium signatures into specific responses. Whilst straightforward as a concept, mysteries remain as to exactly how such information processing is biochemically implemented. Novel developments in imaging technology and genetically encoded sensors (such as calcium indicators), in particular for multi-signal detection, are delivering exciting new insights into intra- and intercellular calcium signaling. Here, we review recent advances in characterizing the encoding, transmission, and decoding mechanisms, with a focus on long-distance calcium signaling. We present technological advances and computational frameworks for studying the specificity of calcium signaling, highlight current gaps in our understanding and propose techniques and approaches for unravelling the underlying mechanisms.

## Teaser

Review summarizing recent technological advances and computational frameworks for studying the specificity of calcium signaling in plant cells.

## Introduction

**Fig. 1.**
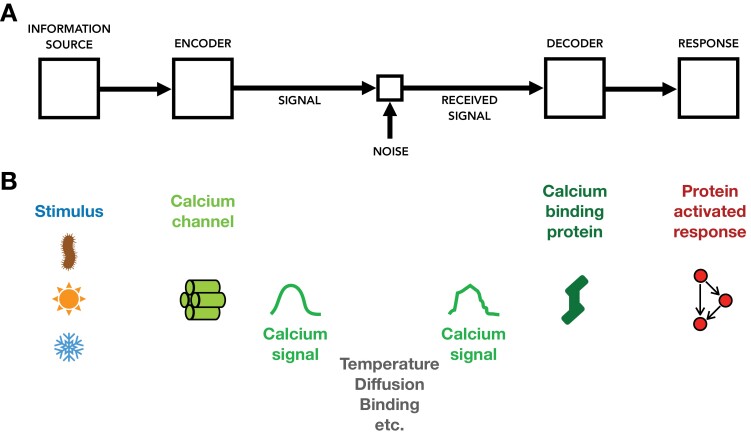
A molecular communications view of calcium signaling. (A) Shannon’s depiction of a communication process. An information source emits messages. The goal of a communication system is to transmit this information to a different location. The messages from the source are encoded into a form that can be processed by the components in the systems (typically binary digits or electromagnetic waves for many technological applications or chemical signals in biology). Due to noise in the system, transmission errors may occur, meaning that the received signal may differ from the sent signal. The goal of coding theory is to develop encoding and decoding algorithms that are robust to such errors (error-detecting and error-correcting codes). (B) Calcium signaling components mapped onto an information processing framework. Environmental cues can be viewed as the information source in this case. These messages are encoded into calcium signals by calcium channels (and associated machinery) and transmitted. Noise may be introduced by physical or chemical factors, such as temperature, diffusion, and binding/unbinding to other molecules in the system. Calcium-binding proteins respond to evaluated free calcium levels and can initiate a response (post-translational, transcriptional). In his mathematical theory of communication, Shannon employed probability theory, and in particular entropy and mutual information, to describe the key characteristics of the system. Recent advances in the field of molecular communications use these approaches coupled with ideas from telecommunications and biophysical principles to describe biological processes such as calcium signaling.

Signaling is critical for plant survival (Kudla *et al.*, 2018; Ackermann and Stanislas, 2020; Cheung *et al.*, 2020; Cheng *et al.*, 2021; Devireddy *et al.*, 2021; Sun and Zhang, 2021; Johns *et al.*, 2021; Klejchova *et al.*, 2021). Responding and adapting to ever-changing conditions requires the ability to sense the surrounding environment and relay this information internally in a form that can action a biochemical change (Lamers *et al.*, 2020). These signaling processes seem analogous to the model of communication or information theory (Shannon, 1948; Box 1; Fig. 1A), and the associated mathematics may provide a useful framework with which to analyse such systems (Harper *et al.*, 2018; Martins *et al.*, 2019; D. Bi *et al.*, 2021).

**Box 1. Information theory**

Information theory describes the process of transmitting a message between locations and/or different times. The message is first put into a form that can be transmitted in a process known as encoding, resulting in an internal signal (for many technical applications, this is a binary representation). Transmission occurs through a physical system that will have inherent noise associated with it (error rates) and limitations on the amount that can be transmitted in any given time (channel capacity). Given noise in transmission, the mapping of the received signal back onto the original message can be prone to errors whenever two signals differ by less than the noise in the system. Information theory provides limits on how much information can be transmitted and with what error-rate. Key concepts of information theory are the frequencies of the elements that make up the signals (the alphabet), characterized by probability distributions, the associated metric for capturing uncertainties, characterized by entropy, and the quantification of relationships between sent and received signals/messages, characterized by mutual information. An astonishing result is that as long as the transmission rate is below the channel capacity, the transmission errors can be arbitrarily small (Shannon, 1948). How to achieve this is the goal of coding theory, which essentially devises algorithms for mapping the original messages onto signals whose distance between permissible code words increases (making the code words more robust to noise), often with built-in error-detection and error-correction features. Further background information can be found in Shannon (1948) and MacKay (2002) and for biological applications in D. Bi *et al.* (2021).

Sensing of environmental conditions typically occurs locally at the cellular level, at the plasma membrane (Ackermann and Stanislas, 2020; Klejchova *et al.*, 2021). Once a signal has been generated upon detection of a stimulus, it needs to be transmitted to the location(s) where a response can be actioned. Local sensing can lead to a local response in the same cell at the same or different subcellular locations. Furthermore, a locally induced signal and response can spread to neighboring cells and allow for systemic signaling to distal parts of the plant (Hilleary and Gilroy, 2018; Liu and Chen, 2018; Farmer *et al.*, 2020; Fichman and Mittler, 2021; Johns *et al.*, 2021). The consequences of such signals include binding events, protein conformational changes and modification, enzyme activation or de-activation, metabolic adjustments, transcriptional regulation, and epi-genetic changes. Some stimuli can be sensed simultaneously in multiple cells, potentially leading to parallel processing of information. Sensing of conditions, internal and external, occurs at all times in cells (Choi *et al.*, 2017; Katano *et al.*, 2018; Chen *et al.*, 2020), raising questions about how different signals are transmitted at the same time, whether they can then still be differentiated and, if so, how these signals are integrated. Despite exciting recent progress in this field (Katano *et al.*, 2018; Suda *et al.*, 2020; Xu *et al.*, 2020; Devireddy *et al.*, 2021; Fichman and Mittler, 2021; Li *et al.*, 2021), the complexity of multiple stimuli, multiple pathways, and signal integration remains poorly understood.

For an agent (molecule, ion) or a physical property of matter (concentration, pressure, stress, voltage) to act as a signal implies (i) a change in this agent or physical property over time in response to a stimulus, (ii) the propagation of this change either to a different location in space and/or to a later time, and (iii) that some action is caused as a consequence of this signal (Morris, 2018). In Shannon’s mathematical theory of communication these processes are known as encoding, transmission, and decoding (Shannon, 1948). If a signal is specific to a certain stimulus, there is a one-to-one mapping of the stimulus to the signal. Hence, if two signals are the same, then the stimulus must have been the same. The speed with which a signal can be altered should reflect the change in the stimulus that the signal is aiming to describe. For instance, signals that are meant to induce responses to insects (fast) will need to be rapid, whereas signals that encode seasonal changes (slow) may not require fast dynamics.

Many different kinds of molecules or ions and some physical properties have been shown to be involved in signaling in plants (Cheng *et al.*, 2021; Fichman and Mittler, 2021; Johns *et al.*, 2021). One of the most versatile and widespread signaling agents in eukaryotes is the calcium ion (Bootman and Bultynck, 2020; Kong *et al.*, 2020; Ambudkar and Hofer, 2020; Luan and Wang, 2021), and calcium ions are key agents for signal transduction in plants (Dodd *et al.*, 2010; Demidchik *et al.*, 2018; Kudla *et al.*, 2018; Tian *et al.*, 2020). Changes in the free cytosolic calcium ion concentration are associated with developmental processes and responses to biotic or abiotic factors in the plant’s environment (Charpentier *et al.*, 2016; Vincent *et al.*, 2017; Zandalinas *et al.*, 2020; Tian *et al.*, 2020).

Given the importance of calcium signaling, its associated machinery has received much attention (Demidchik *et al.*, 2018; Tian *et al.*, 2020; Luan and Wang, 2021; Dong *et al.*, 2021): calcium channels, calcium pumps, and calcium binding proteins have been identified, described, and characterized (biochemically, electrophysiologically, structurally) to varying levels of detail (Kudla *et al.*, 2018). The signal-encoding calcium channels and calcium pumps have been shown to generate a variety of calcium transients in response to external stimuli. Such a spatio-temporal calcium pattern has been referred to as the calcium signature (McAinsh and Hetherington, 1998). The transmission of a calcium signal can be electrical, hydrodynamic, and diffusive (Choi *et al.*, 2017). The decoding of these signals is associated with a large repertoire of calcium binding proteins; for example, Arabidopsis has 1260 genes and 1085 distinct proteins annotated with ‘calcium binding’ (GO:0005509) or with ‘calcium ion sensor activity’ (GO:0061891). These proteins, either directly or indirectly coupled with kinase, phosphatase, or transcription factor activity, can decode calcium signatures into specific responses. Yet, despite tremendous progress in the characterization of components, quantification, and understanding, mysteries remain. Here, we review recent advances in unraveling the encoding, transmission, and decoding of calcium signaling, and present frameworks and technological approaches for studying specificity with the goal of determining the underlying mechanisms.

## Calcium signaling mechanisms and signal specificity

Many different stimuli that plants experience lead to an elevation in the concentration of free (i.e. not bound to other molecules) intracellular calcium ions from their unperturbed resting levels. We shall refer to this change in the free calcium ion concentration in cellular compartments (typically in the cytosol but also the nucleus or other organelles) as a ‘calcium signal’ with the understanding that in several cases the signaling functionality has yet to be demonstrated. Resting, baseline levels of free calcium vary between compartments in the cell, from about 100 to 200 nM in the cytosol (Dodd *et al.*, 2010; Kudla *et al.*, 2018; Tian *et al.*, 2020) and similar levels in the nucleus, chloroplast, and mitochondria, to hundreds of micromolar in the endoplasmic reticulum and several millimolar in the vacuole (Stael *et al.*, 2012; Costa *et al.*, 2018; Resentini *et al.*, 2021a). Calcium signals are used in all eukaryotes, with plants being no exception, and are among the most important internal cellular messengers (Bootman and Bultynck, 2020; Kong *et al.*, 2020; Ambudkar and Hofer, 2020; Luan and Wang, 2021). In this section, we review the models and evidence for different stimuli being encoded in specific calcium signals and for these calcium signals to give rise to specific responses. The general framework we use for describing the calcium machinery is that of information theory, a mapping of which is depicted in Fig. 1A, B.

## Calcium signal encoding

Changes in free calcium ion concentrations arise from the regulation and interplay of calcium channels, calcium pumps, calcium binding molecules (buffers), and calcium stores (Bootman and Bultynck, 2020; Gilabert, 2020). Due to their importance as part of the communication interface with the environment, ion channels that are localized to the plasma membrane have received particular attention. Recent advances include the implication of a role for GLUTAMATE RECEPTOR-LIKE (GLR) calcium-permeable channels in immunity (Bjornson *et al.*, 2021) and the discovery that pathogen-associated molecular pattern (PAMP)-induced calcium signaling relies on two cyclic nucleotide-gated channel proteins, CNGC2 and CNGC4 (Tian *et al.*, 2019). The channels interact with calmodulin in its basal, inactive state. Upon pathogen perception, phosphorylation activates the channels, leading to calcium influx (Tian *et al.*, 2019). Another example is the observation that PAMP perception leads to phosphorylation of the calcium channel OSCA1.3 that is involved in the regulation of stomatal closure (Thor *et al.*, 2020). Intriguingly both these activation events depending on cytosolic kinase activity (BOTRYTIS-INDUCED KINASE1, BIK1). These observations demonstrate the close correlation and association between phosphorylation and calcium signaling. The commonality of some of the kinases and decoding calcium binding proteins that are shared between pathways, however, raises questions about the specificity of these signaling processes.

In addition to the plasma membrane, the importance of other membranes (organelles) is becoming increasingly clear (Costa *et al.*, 2018; Resentini *et al*., 2021*a*, *b*; Grenzi *et al.*, 2021). A key role of nuclear membranes in symbiotic interactions (Capoen *et al.*, 2011; Charpentier *et al.*, 2016) and more recently in root development (Leitão *et al.*, 2019) has been demonstrated. Other exciting breakthroughs include the finding that tonoplast/vacuole voltage plays an important role in calcium signaling (Horaruang *et al.*, 2020; Dindas *et al.*, 2021) for salt stress (Choi *et al.*, 2014; Evans *et al.*, 2016), herbivory (Vincent *et al.*, 2017), and pathogen recognition (Hilleary *et al.*, 2020). Membrane voltage acts as a key integrator of various ionic activities at membranes (Klejchova *et al.*, 2021), providing a natural focus for modeling the generation of calcium signals (Hills *et al.*, 2012; Granqvist *et al.*, 2012; Martins *et al.*, 2016; Jezek and Blatt, 2017; Horaruang *et al.*, 2020).

New advances lead to the integration of the role of membrane contact sites, connecting the ER with chloroplasts, mitochondria, and the plasma membrane. This additional level of communication may facilitate more robust and precise organelle signaling, as well as less dependence on vesicle trafficking. Calcium signaling might rely more than expected on direct communication through a close gap between opposed membranes (Wong *et al.*, 2019). Calcium is also involved in membrane tethering, for example via stromal interaction molecules and the cellular reorganization and movement of organelles through the motor molecules actin and myosin (Tominaga *et al.*, 2012). Interaction and movement of plant cell organelles has been recently the focus of novel developments in plant–microbe interactions research (Perico and Sparkes, 2018; Perico *et al.*, 2021). More and more organelles have been shown to be reorganized during pathogen infection, particularly by *Phytophthora infestans* (Leelarasamee *et al.*, 2018; Savage *et al.*, 2021; Sun *et al.*, 2021). Interestingly, other pathogens have been shown to target motor molecules and calcium binding proteins via effector proteins, modifying these processes.

A perceived change in the environment is often the first event that leads to a calcium signal (Tian *et al.*, 2019; Kong *et al.*, 2020; Thor *et al.*, 2020). For instance, cell surface receptors can bind to ligands that trigger a conformational change that either directly (ligand-activated calcium channel) or indirectly (via changes to the membrane potential, through interactions with a calcium channel or kinases) leads to the activation of a calcium channel. If all the events that follow (diffusion, ATPase activity, buffering, involvement of calcium stores, organelle signaling, etc.) are a consequence of this first calcium transient, then this initial change will need to be unique to the stimulus for the calcium signal to be specific. To test this hypothesis, there is a clear need to determine the calcium transients at high spatio-temporal resolution (Cao *et al.*, 2013), the mechanisms, and the electrophysiological characteristics of calcium channels. If associated signaling events, such as pH (Behera *et al.*, 2018) or reactive oxygen species (ROS) (Evans *et al.*, 2016), are merely downstream consequences of this calcium signal, then no further information would be contained in these pathways, although they may add to the transmission and robustness of the signal.

**Fig. 2.**
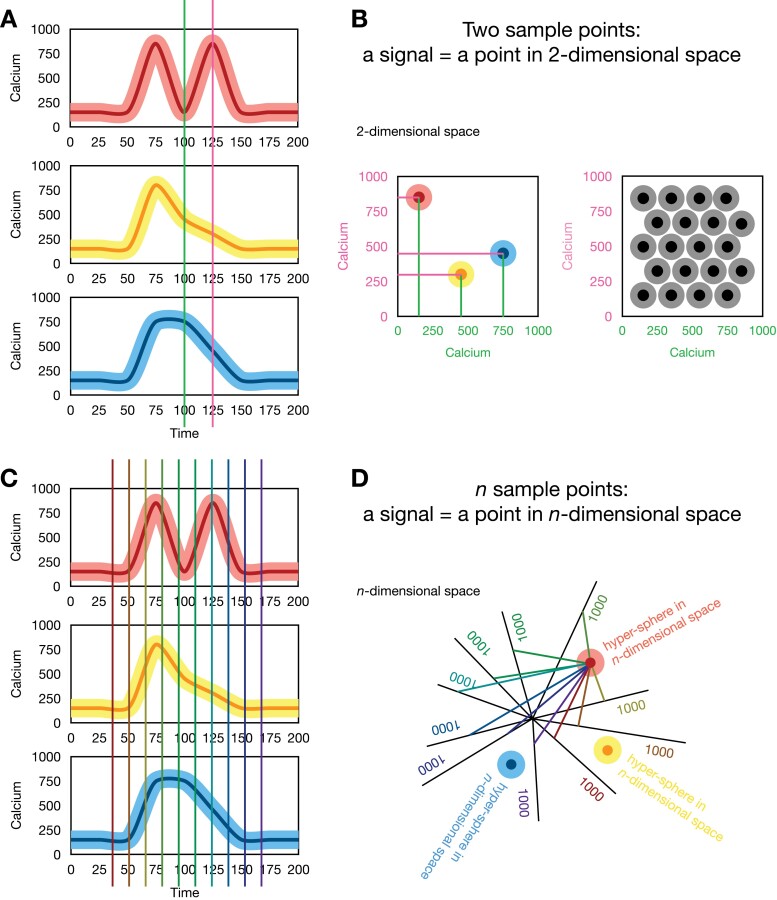
The geometry of calcium signal encoding. (A) For calcium signals to initiate well-defined responses they need to be as distinct as possible. An example of three different calcium signals is shown. To distinguish these signals computationally from another, a metric needs to be defined that allows for the comparison between them (how that is done biochemically is not clear). In this simple example, each calcium signal is sampled at two time points (the green and pink lines). (B) Using two sample points for each calcium signal results in two concentration values, which can be viewed as a point in two-dimensional space. Three calcium signals are depicted as three points in two-dimensional space. The estimated noise/error of the calcium concentration defines a disc of uncertainty around each point (lighter color). As long as these discs do not overlap, the signal will be robust to transmission errors (error-correcting). If each signal is separated in this manner from another signal, then the number of possible distinguishable signals corresponds to the number of such discs (spheres/hyperspheres in higher-dimensional space) that can fit within the range defined by the calcium dynamics. If the discs/spheres were to overlap then the signals could be misinterpreted and lead to an undesired response. An example of how to pack different signals into the space defined by the range of calcium concentrations such that they are robust to noise is depicted for a simple two-dimensional case. (C) Three calcium signals sampled at 10 time points. This generalizes to as many points as one wishes. (D) The 10 (*n*) sample points used to characterize each calcium signal can be thought of as a point in 10 (*n*)-dimensional space and the region of this space that maps to the same signal is a hyper-sphere with a radius defined by the noise level. How proteins might distinguish these signals is touched on in ‘Calcium signal decoding’.

Assuming that the information to be transmitted resides in the calcium signal, then these signals should be as distinct as possible to reduce the risk of errors in transmission or decoding leading to unwanted cellular programs being initiated. For instance, if we describe each signal by two values (say, by the calcium concentration at two time points, or by the amplitude and duration) then each signal can be viewed as a point in two-dimensional space (Fig. 2). If each of these two representative values is subject to the same level of noise, then each signal can be safely identified only if the two signals are always separated by at least two times the expected noise level. In this case, every received message falls within a circle of radius equal to the noise level around a center that corresponds to the error-free signal. Within this approximation, an ideal code would correspond to the optimal packing of discs within an allowable plane of parameter values (Fig. 2). This idea can be extended to however many characteristics of the signal can be evaluated, leading to optimal codes corresponding to sphere-packing in *n*-dimensional space (Fig. 2). Signal amplitude, signal shape, and signal duration can be altered by calcium channels and associated machinery. However, determining how much flexibility exists within biological systems to sample such potential calcium codes remains to be explored.

A key property relating to signal encoding is the capacity of a calcium channel (Eckford and Thomas, 2013, 2018; Thomas and Eckford, 2016), which can reveal how much information can be captured within a given time. Recent progress in this area has led to mathematical models with the associated computational tools for answering such questions (Farsad *et al.*, 2016; Eckford and Thomas, 2018; Martins *et al.*, 2019; Akyildiz *et al.*, 2019; D. Bi *et al.*, 2021). For instance, a framework for computing the channel capacity for a single ligand-activated channel has been derived (Pagliara *et al.*, 2014; Farsad *et al.*, 2016; Thomas and Eckford, 2016; Ratti *et al*., 2020, 2021). It will be important to extend these results to multiple channels to evaluate the overall information transmission rates to gain insights into the encoding process.

## Calcium signal transmission

Local increases in free calcium concentrations will result in directed diffusion down the concentration gradient, leading to transport of the calcium signal. Calcium is, however, highly buffered in cells, so that the movement of calcium binding molecules plays a significant role in the spread of calcium, and the kinetics of binding determines local free calcium concentrations (Sneyd, 1994; Wagner and Keizer, 1994; Smith *et al.*, 1996; Granqvist *et al.*, 2012; Gilabert, 2020). A consequence of strong buffering within a crowded cellular environment is a large drop in the effective diffusion of calcium. Molecular diffusion will lead to a gradual flattening of the initial calcium signal, raising the question of how signal specificity can be maintained. Furthermore, the diffusion time scales quadratically with distance, which coupled with small diffusion constants makes this mode of transport ill-suited for anything beyond sub-cellular domains (Falcke, 2004).

Diffusion alone is too slow for long-distance signaling but can be effective when coupled with other mechanisms. Spatiotemporal calcium waves in frog eggs have been modelled using the fire–diffuse–fire framework (Dawson *et al.*, 1999). In this model, a calcium channel is activated (fire) and this event causes (via short-range diffusion) the next channel to be activated (fire), giving the impression of a traveling calcium wave. This framework has been used to model the spread of calcium oscillations around the nucleus and between the cytosol and nucleosol (Capoen *et al.*, 2011; Martins *et al.*, 2016). Even with the fire–diffuse–fire mode of signal propagation, long-distance signaling speeds could not be recapitulated with biophysical parameters that were consistent with experiments (Evans *et al.*, 2016). This led to the development of a mathematical model of coupled waves between reactive oxygen species and calcium (Evans *et al.*, 2016), which was based on a previously suggested conceptual model of ROS and calcium interactions (Dubiella *et al.*, 2013).

**Fig. 3.**
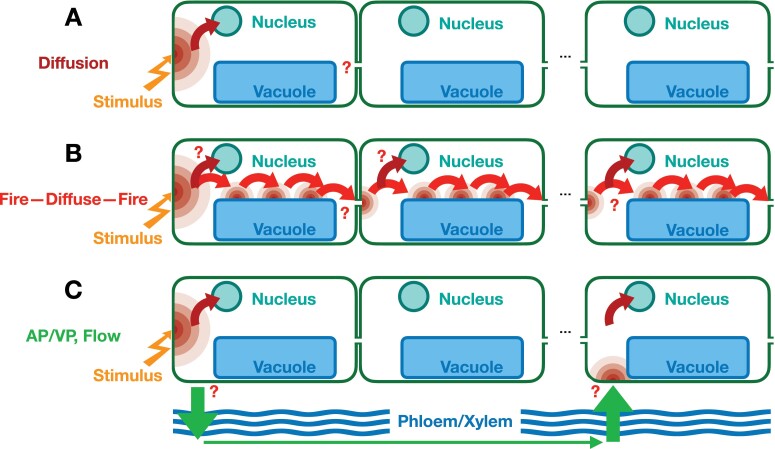
Transmission mechanisms for calcium signaling. (A) Any generated calcium signal will spread out through molecular diffusion. This mode of signal transmission is most effective over short distances. How useful diffusion alone is for the transmission of intercellular signals remains to be determined. (B) The combination of diffusion with the mechanism, either directly or indirectly, of calcium-induced calcium-release allows for the generation of calcium waves. ROS waves may be coupled to this process. (C) The vasculature is used for rapid long-distance signaling, by electrical mechanisms (action potentials, AP; variation potentials, VP), bulk flow (VP), or potentially pressure waves (VP). Many questions remain regarding the detailed mechanisms.

Other mechanisms of long-distance transport that are associated with calcium include action potentials and variation potentials (Choi *et al.*, 2017; Fichman and Mittler, 2021; Johns *et al.*, 2021; Mudrilov *et al.*, 2021). Models of the actual signal propagation mechanism are based on electric potentials (Hedrich *et al.*, 2016; Vodeneev *et al.*, 2016; Sukhov *et al.*, 2019; Mudrilov *et al.*, 2021; Sukhova *et al.*, 2021), chemical transport (Sukhov *et al.*, 2013; Evans and Morris, 2017; Blyth and Morris, 2019), or pressure (Malone and Stanković, 1991; Farmer *et al*., 2014, 2020; Moe-Lange *et al.*, 2021), rather than long-distance movement of calcium (Fig. 3). The term ‘hydraulic wave’ is often used in the context of wounding responses and seems to refer to two very different phenomena, namely bulk flow driven by a pressure differential or propagation of a pressure disturbance (sound wave) without actual transport of material (Malone and Stanković, 1991; Evans and Morris, 2017). The xylem is under tension and the release of this tension through wounding would likely result in a near-instantaneous equilibration of pressure (Farmer *et al.*, 2020). The evidence for the involvement of mechano-sensitive channels in long-distance signaling is accumulating, which could support a pressure wave (pressure increase), bulk flow (pressure decrease), or subsequent osmolyte changes. The question of how such transmission mechanisms can maintain the presumed specificity of a calcium signal remains to be resolved.

## Calcium signal decoding

**Fig. 4.**
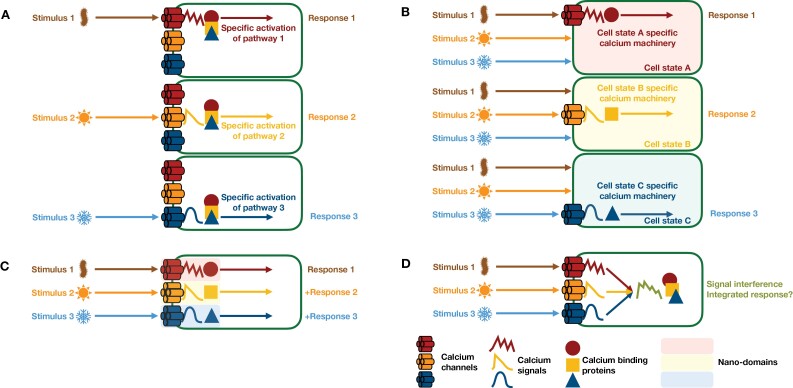
Concepts for the establishment of specificity in calcium signaling. (A) Stimulus specificity resides in the calcium signal. In this scenario, every stimulus is encoded into a calcium signature that activates only those proteins and pathways that are required to initiate the appropriate response. This model requires that there is a one-to-one mapping between each stimulus and each calcium signature, i.e. there is a unique calcium signature for every stimulus. Furthermore, decoding of each unique calcium signature must lead to the activation of the target protein, such that other calcium binding proteins are not significantly activated. (B) Signal specificity is a function of the cell state. In this scenario, only certain cell-types or cells in certain states can respond to the stimulus. This could be achieved by cell-type/cell-state specific expression of genes. In this model, there is no need for specificity in the calcium signal itself. The calcium signals could, in principle, all be the same and their interpretation would be a consequence of the available proteins in the cell. (C) Signal specificity depends on local nano-domain composition. This scenario is analogous to (B) but at the spatial resolution of nano-domains rather than cells. As long as the encoding and decoding machinery are co-located in the same nano-domain there would be no requirement for signal specificity to reside in the calcium signal. (D) Signal interference. Under the scenario outlined in (A), multiple stimuli could lead to interference between various calcium signals. To what extent this occurs and how this could be untangled remains unclear.

The calcium signal hypothesis suggests that the spatio-temporal dynamics of free calcium ions represents an encoded stimulus (McAinsh and Hetherington, 1998). Such calcium signatures can be decoded by the appropriate calcium machinery (Larsen *et al.*, 2004; Hashimoto and Kudla, 2011; Miller *et al.*, 2013; Lenzoni *et al.*, 2018; Poovaiah and Du, 2018). Differences in the proteome of cell types, potential cell clusters, and organs will determine how cells receive, transduce, and translate a signal (Fig. 4); for example, the presence or absence of stimulus-specific calcium channels will change how cells respond. Thus, there is more to decoding than the signatures of the code. The function of calcium as a signal is inseparably intertwined with calcium binding proteins, calcium channels, and transporters. As mentioned above, the latter are of key importance for the formation of specific signatures in response to internal changes or cues in the environment, whereas calcium binding proteins allow for transduction and translation of the signal. In all cases, cell specific expression patterns are likely to contribute to a functional and cell specific signaling and decoding. Calcium-binding proteins characteristically contain an EF-hand motif, responsible for calcium binding, and examples include calmodulin (CaM), CaM-like (CML), calcium-dependent protein kinases (CDPK), and calcineurin B-like (CBL) proteins (Dodd *et al.*, 2010; Kudla *et al.*, 2018; Tang *et al.*, 2020; Dong *et al.*, 2021).

Many calcium responders and decoders have been identified and several have been kinetically characterized, but there are only a few documented cases for which a decoding mechanism has been described in plants (Miller *et al.*, 2013; Lenzoni *et al.*, 2018; Martins *et al.*, 2019). It is assumed that calcium binding proteins can be activated by a calcium-induced conformational change. Microscopic rate constants for calcium binding are typically rapid compared with transcriptional or post-translational steps. This means that if calcium decoders were based only on calcium binding events, to a good approximation, they would simply follow calcium dynamics (Swainsbury *et al.*, 2012). Coupling this to a slower process, such as phosphorylation, allows decoder proteins to integrate calcium signals and to retain a memory from the activation (Miller *et al.*, 2013). Recent breakthroughs that support a memory effect of calcium include the identification of calcium-triggered activation of Venus’ fly trap based on previous recognition events (Suda *et al.*, 2020) and the activation of stomatal dynamics based on previous photosynthetic activity (Jezek *et al.*, 2021).

A significant breakthrough on the design principles of a calcium communication system, and in particular the decoding process, has recently been described (Liu *et al.*, 2020). Previously, the authors provided strong experimental evidence that different calcium signals result in different transcriptional responses (Lenzoni *et al.*, 2018). In their recent important contribution, they provide mathematical models that show how transcription factor levels can change as a function of the fourth power of the change in calcium concentration. This effect can lead to several orders of magnitude differences in activated transcription factor concentrations. Thus, as long as the input signals are sufficiently different, this provides a theoretical explanation for how specificity may be achieved.

## Technological advances

Quantifying real-time spatiotemporal calcium dynamics is one of the most important steps towards understanding calcium signaling in living organisms (Grenzi *et al.*, 2021). Technological advances over the past decade include multiple detection methods for calcium imaging in living cells, such as calcium binding, visible-light-excitable fluorescent dyes and genetically encoded calcium indicators (GECIs) (Zhou *et al.*, 2021). These developments are delivering new insights and quantification at increasing spatio-temporal resolution with multiple sensors simultaneously. Some related challenges in relation to interpreting calcium images have been described (Vaz Martins and Livina, 2019). Excellent reviews on the latest imaging advances have recently been published (Clark *et al.*, 2020; Grenzi *et al.*, 2021; Rowe and Jones, 2021; Sadoine *et al.*, 2021).

Chemical dyes for the detection of calcium are either dynamic single-wavelength light-excitable or ratiometric indicators. Single-wavelength indicators include Oregon Green 488 BAPTA-1, and Fluo-4 and Fluo-5 acetoxymethyl ester and pentapotassium, each containing a fluorophore and calcium chelator, with increased dynamic range for qualitative analysis (Kanchiswamy *et al.*, 2014; Lock *et al.*, 2015). Limitations include variability in determining intracellular calcium levels as a consequence of dye extrusion, uneven loading, dye retention, photobleaching, and cell toxic side effects (Gasterstädt *et al.*, 2020; Li and Saha, 2021). Ratiometric or dual-wavelength dyes are UV-excitable, and quantitatively detect fluctuations in target ion concentrations. Ratiometric calcium indicators include Fura-2 acetoxymethyl ester, Fura-Red acetoxymethyl ester, Fura-2 pentapotassium, Indo-1 acetoxymethyl ester, and Indo-1 pentapotassium (Tinning *et al.*, 2018). These dyes exhibit a shift in emission or excitation spectrum upon calcium binding (Tang *et al.*, 2021). Utilizing ‘ratio’ technology yields a more accurate readout for quantitative real-time measurements, although such chemical treatments are not suited for long-term imaging.

Genetically encoded calcium indicators or dual-Förster resonance energy transfer (FRET)-based sensors track real-time and long-term calcium signaling. Advantages of single fluorescent sensors are high dynamic range and tapered excitation and emission range optimal for application of dual-fluorescent molecules for simultaneous imaging without the need for cofactors (Suzuki *et al.*, 2016). The first protein-based chemiluminescent calcium sensor was aequorin, isolated from the *Aequorea victoria* jellyfish (Knight *et al.*, 1991). Advances in biosensor technology led to single fluorescent protein (FP)-type sensors, consisting of a calcium responsive element and circularly permutated FP that undergoes a conformational change upon calcium binding, altering the protonation of the FP fluorophore (Chen *et al.*, 2017). Nonetheless, using a single emission range for detection can be limiting, leading to changes in sensor expression levels potentially being misinterpreted (Zhong and Schleifenbaum, 2019). Such calcium indicators include GCaMPs, a fusion products of green fluorescent protein (GFP), CaM, and the peptide sequence M13 from myosin light chain kinase with circularly permutated enhanced GFP and a CaM domain with four EF-hand calcium binding motifs (Kostyuk *et al.*, 2019). Upon calcium binding, conformational changes alter fluorescence intensity—due to CaM-induced modification of GFP (Akerboom *et al.*, 2012). Development of GCaMP1 in transgenic mouse models showed reduced background noise but unstable fluorescence (X. Bi *et al.*, 2021). Technological advances produced GCaMP2–3, GCaMP5–6, and jGCaMP7, exhibiting increased fluorescence, stability, signal-to-noise ratio, dynamic range, and responsiveness (X. Bi *et al.*, 2021). Plant constructs such GECOs (R-GECO, G-GECO) have recently been implemented in Arabidopsis (Keinath *et al.*, 2015; Waadt *et al.*, 2017). Nonetheless, recording fast fluorescence transients in neurons and plant signaling requires fine-tuned spatio-temporal resolution, limited by the kinetics of GECS. Fast and slow variants of GCaMP6 and jGCaMP7 could circumvent this, whereby high sensitivity indicators with slow kinetics produce strong fluorescence signals, whereas low sensitivity indicators with fast kinetics can capture signal dynamics in more detail.

Dual-FP sensors contain a calcium responsive element linked by two FPs, governed by FRET. FRET is a mechanism of energy transfer between two light sensitive molecules (Suzuki *et al.*, 2016). Energy transfer occurs only if the distance between fluorophores with overlapping emission and absorption spectra falls below about 10 nm and the dipole orientation is appropriate (Marx, 2017). An advantage of dual-FP sensors is that they produce a ratiometric readout for more accurate interpretation of data, but they are limited in their detection range, dynamic range, and signal reduction due to FP barrel size and rotatable peptide linkers (Depaoli *et al.*, 2019). Cameleon was the first and most widely utilized dual-FP sensor, structurally composed of calmodulin and M13 from myosin light chain kinase peptide linked to cyan and yellow FPs (Miyawaki *et al.*, 1997; Germond *et al.*, 2016). Recently, increased dynamic range of single-FP GECOs was combined with ratiometric readouts, yielding the Matryoshka sensor, containing a circularly permuted green FP functioning as a receptor and a nested stable large Stokes shift LSSmOrange internal control FP, for a single excitation overlap at 440 nm, producing green and orange emission for ratiometric readout (Ast *et al.*, 2017). Similarly, R-GECO1-mTurquoise retains similar features to single-FP biosensors but still harbors a reference FP fused to several single-FP intensiometric GECIs (Waadt *et al.*, 2017). Both cassettes can quantify cytosolic calcium level in plant roots. Such technological advances may be the backbone of future biosensor engineering. The most recent progress in calcium sensor technology was the development of increased 4-fold ultra-fast and 3.5-fold more sensitive jGCaMP8 varieties (fast, medium, selective), broadly tested in mammalian neurons *in vitro* and *in vivo* (Grødem *et al.*, 2021, Preprint).

An exciting recent advance is the development of CapHensor (Li *et al.*, 2021), an optimized dual-reporter for the simultaneous imaging of calcium and pH. Using this technology, the authors determined the spatio-temporal calcium dynamics in pollen tubes, guard cells, and mesophyll cells and were able to gain new insights into the connections between calcium, pH, and membrane voltage changes (Li *et al.*, 2021). Such developments are key for unravelling the complexities and contributions of multiple variables to the encoding and specificity of signals.

Recent research has expanded on a pre-published unidirectional dual-flow root chip (dfRC) (Stanley *et al.*, 2018) microfluidic platform, adding bi-directional flow capabilities to visualize and quantify osmotic stress on the plant root system. The root-chip is combined with plants containing a fluorescent calcium detector and the corresponding signals can be visualized by fluorescence microscopy (Grossmann *et al.*, 2011; Clark *et al.*, 2020; Sadoine *et al.*, 2021). This novel technology provides a unique opportunity to challenge the root with two different conditions simultaneously, and observe signal transduction, root growth (and force), and local adaptation processes at the same time under adaptable environmental conditions.

## Discussion

A question often posed is how calcium ions can encode so many different signals. Our verbal communication (language) also relies on a single entity changing in space and time: through fluctuations in the local concentration of air molecules and the associated pressure changes, we can transmit seemingly endless amounts of data, noise and information through sound. Thus, it is certainly conceivable that local changes in the concentration of other molecules, or ions such as calcium, could convey a similar multitude of information. So, one entity being able to produce many signals is perhaps not so puzzling. However, the corollaries of this statement remain open question. We still do not know exactly how different calcium signals are encoded, how different they are, whether there is a one-to-one mapping from stimulus to calcium signal, how the signals are transmitted, whether information is lost during transmission, and how the signals are decoded. Although calcium is a universal messenger, involved in a vast array of important processes and with hundreds of identified involved proteins, we actually know rather little in terms of precise mechanisms and predictive power.

Keeping to the language analogy, non-verbal communication (‘body language’) is considered to be at least of equivalent importance to verbal language, and many authors state that it is even more important. Whilst those statements can be questioned and without proper quantification would be difficult to assess, there is no doubt that non-verbal communication can provide an additional source of information. For example, if our collaborator told us that they are passionate about calcium signaling and of course they would work 100% on our project but coupled this statement with a big yawn after ‘calcium’, breaking eye contact, and fidgeting with a pen, this would not necessarily strengthen the information they might have wished to convey. Our own error-detecting decoder may note this additional information, which may affect our behavior and decision making. Perhaps analogously, non-calcium communication may play a role in transmitting additional information that may act to support, reinforce, or counter the information in the calcium signal. Another possibility is that it is these non-calcium signals that convey specificity and calcium acts more akin to an alarm system (‘something is wrong’). Recent results have been reported that document calcium-associated pH changes, ROS signals, membrane voltage changes, and hormones. The development of biosensors that can simultaneously monitor different signals is a significant advance in this context that may allow for different types of signals, their correlations, and differences to be determined for different stimuli. A follow-up question is then how this information can be robustly decoded, and related to this is the question of specificity.

Despite decades of research, the mechanistic details of how calcium signal specificity is established remain to be elucidated. How can calcium activate specific pathways but not also other calcium-induced pathways? Does every stimulus have its own channel to encode into a calcium signal? The expansion of the families of calcium channels in mammals is consistent with the link between a stimulus and a specific calcium signal; the reduction of the number of channels in plants seems not to be. Yet, the number of potential calcium signal decoding proteins in plants is vast. Should not the complexity of encoding and decoding be matched?

If information resides in the spatiotemporal pattern of calcium, then this information will need to be either maintained directly in the calcium signal or somehow otherwise encoded in the transmission process. How this might happen is not clear. If specificity is not encoded in the calcium signal, then the question arises as to where this comes from. ROS, pH, and electrical signals are excellent candidates for sources of additional information and have been observed during calcium signaling. Do these events occur upstream of calcium changes? Are they interlinked? For these signals to carry additional information, however, necessitates some level of independence from the calcium signal as they would otherwise be highly correlated and not add anything. The possibility of determining individual cell states, subcellular calcium signals, and transcriptional networks for single cell types for different perturbations offers the highly exciting opportunity to gain further, detailed insight into this important signaling system. Recent progress in modelling calcium pathways and decoding processes, and in particular advances in the field of molecular communications, coupled with increased spatio-temporal imaging may shed light on this long-standing problem.

## Conclusions

Significant recent progress has been made in dissecting the information processing components of calcium signaling in plants. Much is now known about the calcium machinery, including kinetic and electrophysiological characterization. Exciting new results include the identification and characterization of mechano-sensitive channels that are required for the transmission of calcium signals and/or read-out of other signals during long-distance signaling, the development of computational frameworks for studying calcium signaling and in particular advances on decoding mechanisms, and the identification of other signals (e.g. ROS, pH, electrical signals) that co-occur with calcium. A particularly promising advance is the development of tools that allow for the simultaneous *in vivo* imaging of calcium in different subcellular compartments, such as the cytosol and the ER.

Key to unravelling the details of calcium signaling is the ability to detect, image, and quantify calcium changes and to relate these to various stimuli and the downstream responses. To unpick the various hypotheses for specificity presented here will require sub-cellular resolution imaging of calcium and other entities combined with single-cell transcriptomics and proteomics to determine local changes in gene regulatory networks. Further information on cell-type-specific available calcium machinery associated to developmental stage, as well as fast responses in the form of post-translational modifications will be key to better understanding signaling and decoding mechanisms. The identification of the associated cellular machinery and its kinetic characterization (calcium binding kinetics, protein–protein binding, post-translational dynamics such as phosphorylation rates, transcription factor binding, transcription rates, etc.) will then allow linking calcium signatures and these downstream events to be modeled and understood mechanistically at the molecular level.

**Fig. 5.**
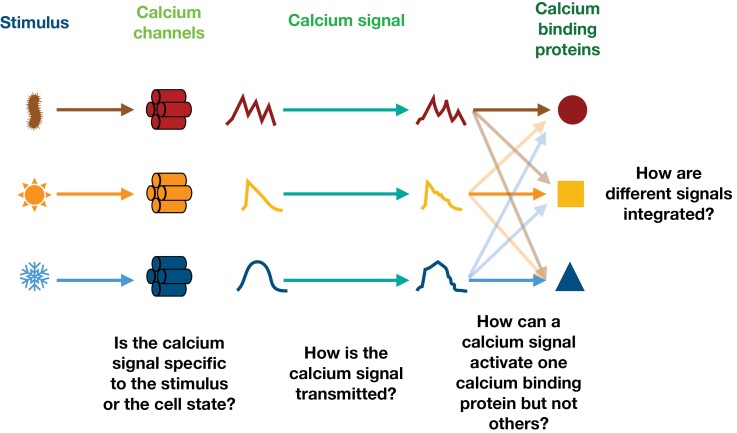
Open questions in calcium signaling. The identity and mechanism as well as the biochemical, kinetic, and electrophysiological characterization of many calcium channels and associated calcium machinery remain to be determined. Furthermore, how these channels interact with or are modulated by other components is poorly understood. How are different stimuli encoded into different signals? Are they specific? What information is actually encoded? What determines which pumps are activated to shape the calcium signal? Are nano-domains important for specificity and what is their composition? What is the mode of signal transmission and what components are involved in this process? How do these signals maintain specificity during transmission? Whilst it is relatively well understood how calcium signals may differentially activate different proteins based on their binding kinetics, it remains unclear how specificity can be implemented biochemically such that each signal maps to one response (without activating others). How signals are integrated, both different calcium signals but also the combination of calcium with other signals, is poorly understood.

Open questions include the following (Fig. 5): 

What information about the stimulus is encoded in the calcium signal? Presence of a stimulus? Nature of that stimulus? Concentration or intensity of the stimulus?
What is the rate at which information can be encoded (channel capacity)?
What are the mechanisms for maintaining the information content of a stimulus-specific calcium signal during transmission?
How are calcium signals decoded such that only the appropriate responses are initiated (specificity)?

## Data Availability

No data were generated for this review.
